# Sexual violence against female university students in Ethiopia

**DOI:** 10.1186/s12914-017-0127-1

**Published:** 2017-07-24

**Authors:** Yohannes Mehretie Adinew, Mihiret Abreham Hagos

**Affiliations:** 1 College of Health sciences and Medicine, Wolaita Sodo University, Sodo, Ethiopia; 2 School of Education and Behavioral sciences, Wolaita Sodo University, Sodo, Ethiopia

**Keywords:** Sexual violence, Sexual harassment, Rape, Female students, Ethiopia

## Abstract

**Background:**

Though many women are suffering the consequences of sexual violence, only few victims speak out as it is sensitive and prone to stigma. This lack of data made it difficult to get full picture of the problem and design proper interventions. Thus, the aim of this study was to assess the prevalence and factors associated with sexual violence among female students of Wolaita Sodo University, south Ethiopia.

**Methods:**

Institution based cross-sectional study was conducted among 462 regular female Wolaita Sodo University students on April 7/2015. Participants were selected by simple random sampling. Data were collected by self-administered questionnaire. Data entry and analysis was done by EPI info and SPSS statistical packages respectively. Descriptive statistics were done. Moreover, bivariate and multivariate analyses were also carried out to identify predictors of sexual violence.

**Result:**

The age of respondents ranged from 18 to 26 years. Lifetime sexual violence was found to be 45.4%. However, 36.1% and 24.4% of respondents reported experiencing sexual violence since entering university and in the current academic year respectively. Life time sexual violence was positively associated with witnessing inter-parental violence as a child, rural childhood residence, having regular boyfriend, alcohol consumption and having friends who drink regularly; while it was negatively associated with discussing sexual issues with parents.

**Conclusion:**

Sexual violence is a common phenomenon among the students. More detailed research has to be conducted to develop prevention and intervention strategies.

**Electronic supplementary material:**

The online version of this article (doi:10.1186/s12914-017-0127-1) contains supplementary material, which is available to authorized users.

## Background

Violence against women includes any act of gender based violence that results in physical, sexual, and psychological harm to women [1]. Any sexual act or attempt, unwelcomed sexual advances, against a person’s sexuality using force, by any person regardless of their relationship to the victim, in any setting is considered sexual violence [2, 3].

Sexual violence is one dimension of violence in schools which creates an atmosphere of intimidation and danger in an environment [4]. It is a fundamental violation of human right to liberty and freedom from fear, and is now recognized as a public health priority [5]. It ranges from forcible rape to physical forms of pressure that compel women to engage in sex against their consent. It is common among children, adolescents and women both in industrial and developing nations [6, 7].

Studies showed that violence against girls by older male students and teachers is very common [8–10]. Women’s suffer physical, mental and reproductive health consequences of sexual violence like depression, loss of self-confidence, injuries, unwanted pregnancy, sexually transmitted diseases and disability up to death [11, 12]. Rape alone results in about 32,000 unwanted pregnancies each year globally [13]. This can have an emotional impact and is also linked to negative health behaviors, such us substance use and mood disorders like anxiety and depression [14, 15].

Violence against women is major public health and human rights agenda in Ethiopia. However, the magnitude of sexual violence among youths is not deeply recognized. Therefore, determining the magnitude and identifying predictors helps to design prevention and controlling strategies to tackle it. Thus this study was aimed at assessing sexual violence and its predictors among female students of Wolaita Sodo University, Ethiopia.

## Methods

### Study design and area

Institution-based cross-sectional study was employed among female Wolaita Sodo University students. The University is located in Wolaita Ethiopia, 327 km to the south of the capital Addis Ababa. More than eleven thousand under graduate regular students were in the university.

### Sample size and sampling procedure

Single population proportion formula was used to determine sample size with the assumption of: 24.2% [16] proportion of attempted rape, 95% confidence level and 4% margin of error. 10% was added for possible nonresponse, making the final sample size 484. Samples were allocated to all schools and colleges proportion to size. Female students were categorized in three strata as first, second, and third year and above, then recruited by simple random sampling technique from the frame provided by the university registrar. Selected students were contacted through their respective departments and were oriented about the study and their random selection. Appointment was made for the day of data collection after deep discussion that removed their doubts and cleared their confusions.

### Data collection tools and procedures

A structured self-administered anonymous questionnaire, adapted with some modifications from the WHO multi-country study on women’s health and life events [10, 17] and other related study [13] was used (Additional file 1). The questionnaire was translated to Amharic and then back to English by different language experts to check for internal consistency. Data collection was facilitated by nine enumerators and one supervisor on April 7, 2015. Respondents filled the questionnaire simultaneously in nine lecture halls.

### Data processing and analysis

After checking for completeness the filled questionnaires were entered into EPI info and analyzed by SPSS. Descriptive statistics were done. Both bivariate and multivariate logistic regression models were also carried out. Odds Ratios and their 95% confidence intervals were computed and variables with *p* value <0.05 were considered significant.

### Data quality assurance

Data quality was maintained by giving training and appropriate supervision for the data collectors. Pre-test was conducted on 25 female students of Wachemo University and proper modifications were made to the tool. By conducting repeated revisions, the questions were made as simple as possible to be answered by the students.

### Study variables [9, 10, 13, 17]

The outcome variable of the study was **s**exual violence whereas the predictors were socio-demographic characteristics, sexual history, behavioral attributes and family history.

### Operational definitions [3, 5]

**Sexual Violence:** acts that are done on a woman by coercion, intimidation or threatening to have sex or engage in acts of sex without the girl’s will. It includes sexual harassment, attempted rape and rape.

**Rape:** any non-consensual penetration of the vagina, penetration obtained by physical body harm, by threatening or deception or when the victim is unable to give consent.

**Attempted rape:** a trial to have sex without consent by coercion, by threatening or deception or when the victim is unable to give consent but without actual penetration of the vagina.

**Sexual harassment:** unwanted sexual behaviors including jokes, verbal comments and physical contacts that are intentionally done on women or girls.

**Witnessed inter-parental violence as a child:** if the respondent had ever witnessed physical violence between her parents or adults who raised her, before age of 14.

## Results

### Socio-demographic characteristics of study participants

Out of the expected 484 respondents, 473 agreed to participate. But, 11 questionnaires were incomplete and discarded. So, full response was obtained from 462 participants yielding a response rate of 95.4%. The median age of respondents was 20 years. More than half of respondents (57.4%) were between the ages of 18 and 20 years. Among all, 43.7% of the respondents were Orthodox Christians, while 23% of the participants were from Wolaita ethnic group. Almost all (98.5%) of the study participants were single and around half (52%) of the respondents were from rural areas (Table 1).

**Table 1:** Socio-demographic characteristics of Wolaita Sodo University students, 2015, *N* = 462

Variables	Frequency	Percentage %
Age		
Median 20.00, Range 18–26		
Religion		
Orthodox	203	43.9
Protestant	146	31.6
Muslim	91	19.7
Others	22	4.7
Place where they came from		
Rural	240	52.0
Urban	222	48.0
Ethnic origin		
Wolaita	107	23.1
Amhara	102	22.0
Oromo	74	16.0
Hadiya	40	8.6
Tigre	31	6.9
Others	108	23.3
College/school		
Engineering	138	29.9
Natural & computational sciences	83	18.0
Agriculture	52	11.3
Business and economics	43	9.4
Social science and humanities	41	8.9
Health sciences	35	7.5
Education and behavioral science	33	7.1
Law	21	4.5
Veterinary medicine	16	3.4
Year of study		
Year I	223	48.3
Year II	147	31.8
Year III+	92	19.9

### Substance-use and related behaviors

Regarding substance use, 15% admitted chat chewing, while 33 (7.1%) and 253 (54.7%) of the respondents testified cigarette smoking and drinking alcohol some day in their life respectively. Fifty-one (11%) of the participants said that they were drunken some day in their lifetime. About quarter (22.5%) of the respondents witnessed they had either male or female close friends who drink regularly.

### Sexual experiences

Among the total respondents, 291 (62.9%) disclosed that they had experienced sexual intercourse. Fifty five (18.9%) of them started sex before the age of 15, whereas 225 (77.3%) started between the ages of 15 and 17 years. Among those who had sexual experience, 22.9% of them reported that they have experienced more than one sexual partner in their lifetime; while 23 (5.6%) of them said that they have more than one sexual partner at the time of study (Table 2).

**Table 2:** Sexual experiences of Wolaita Sodo University students, Ethiopia, 2015, *N* = 462

Variables	Frequency	Percentage %
Ever had sexual intercourse (*n* = 642)		
Yes	291	62.9
No	171	37.1
Age at first sexual intercourse (*n* = 291)		
< 15 Years	55	19.0
15–17 Years	225	77.3
> 18 Years	11	3.7
Age of first sexual partner (*n* = 291)		
< 18 Years	3	1.0
18–24 Years	54	18.6
> 24 Years	187	64.3
Do not know	47	16.1
Did you have prior sexual experience when you first encounter sexual violence?(*n* = 291)		
Yes	183	63.0
No	108	37.0
Number of sexual partners in lifetime (*n* = 291)		
One	222	76.3
Two	38	13.0
Three	18	6.2
Four and above	13	4.5
Number of sexual partners currently (*n* = 291)		
Only one	263	90.4
More than one	28	9.6

### Sexual violence

Lifetime prevalence of any form of sexual violence was reported by 210 (45.4%); while violence in the current academic year and since joining university was reported by 113 (24.4%), and 167 (36.1%) respondents respectively. Students who faced sexual violence before joining university were 182 (39.5%). Among the 45.4% who experienced sexual violence, lifetime prevalence of rape was 71 (15.3%), while since joining university and in the current academic year were 37 (8%) and 11 (2.3%) respectively. Sexual harassment and attempted rape in the current year were reported by 75 (16.2%) and 70 (15.2%) respondents respectively.

### Trend of reporting sexual violence to anyone else

Among the 71 rape cases, 55% faced it once while 9.8% faced it four & above times. Nearly one fifth (19.7%) of the rape cases were informed to family while only 8.4% cases were brought to police (Table 3). Sexual violence was thought as a major problem by 62.1% of the respondents. But 21.9% believed it is not preventable and 19% didn’t know it is a crime. More than one third (35.5%) of the respondents were not getting any information regarding sexual violence.

**Table 3:** Victim students’ frequency and tendency of reporting rape to anyone, 2015 (*n* = 71)

Variables	Frequency	Percentage %
Frequency of facing rape in life time		
One time	39	55.0
Two times	13	18.2
Three times	12	17.0
Four times or more	7	9.8
Family shared (knew) about the rape		
Yes	14	19.7
No	57	80.3
Rape applied/reported to the legal system or police		
Yes	6	8.4
No	65	91.6
Reasons for not sharing/telling to anybody about the rape^a^		
Feeling of shame/guilty	39	54.9
Afraid of families reaction	28	39.4
Didn’t know what to do	26	36.6
Afraid of the public reaction	14	19.4
Afraid of the perpetrator	11	15.4
Other	7	9.8

^a^Multiple responses possible, cannot add up to 100%

### Perpetrators of sexual violence

Most (85%) of the rapists were known to the victims***.*** More than half 41 (57.7%) of the rapists were intimate partners whereas family members/other relatives accounted for 16 (22.5%). Strangers and teachers equally contributed to the 10 (14%) of the 71 rape cases while students alone contributed 4 (5.6%).

### Factors associated with sexual violence

Being in the age category of 20–24, being from rural area, witnessing inter-parental violence as a child, having regular boyfriend, alcohol consumption and having friends who drink on regular basis were found to have positive association with life time sexual violence; while discussing sexual issues with parents showed negative association.

Students with rural childhood residence were about 2 times [OR: 2.51; 95% CI: 1.03, 2.12] more likely to report sexual abuse than those from urban. Students in age group between 20 to 24 years were about 2 times more likely to be victim of sexual abuse compared to the older (>24 years category) [OR: 2.09; 95% CI: 1.23, 3.01] ones. Respondents who had witnessed inter-parental violence during childhood were almost 2 times more likely to face sexual abuse compared to their counterparts [OR: 1.98; 95% CI: 1.82, 3.12]. The odds of sexual abuse is around two times [OR: 1.89, 95% CI: 1.42, 2.99] higher among those who had regular boyfriend in the past or at the time of the study. Similarly, students who drink or have history of drinking [OR: 2.55, 95%CI: 1.08, 2.52] and reported to have a friend who drinks regularly (be female or male) [OR: 3.01, 95% CI: 2.87, 3.91] reported more sexual violence than those who have never consumed alcohol and have no peers who regularly consume alcohol respectively. Moreover, the likelihood of experiencing sexual violence among students who did not discuss personal affairs with parents increased by 74% [AOR: 0.26, 95% CI: 1.40, 3.56] than those who discuss (Table 4).

**Table 4:** Bivariate and multivariate logistic regression analysis output of factors associated with sexual violence among Wolaita Sodo University students, Ethiopia, 2015

Variable	Sexual violence		OR (95% CI)	
	Abused	Not abused	COR	AOR
Age group				
> 24 Years	14	32	1.00	1.00
20–24 Years	103	86	2.73 (1.37–5.40)	**2.01 (1.23–3.01)**
< 20 Years	93	134	1.58 (0.79–3.09)	1.12 (0.69–2.61)
Childhood residence				
Urban	69	153	1.00	1.00
Rural	141	99	3.45 (2.34–5.03)	**2.51 (1.03–2.12)**
Witnessed inter-parental violence as child				
No	86	173	1.00	1.00
Yes	124	79	3.15 (2.14–4.61)	**1.98 (1.82–3.12)**
Do you have a regular boyfriend?				
No	58	113	1.00	1.00
Yes	152	139	2.13 (1.44–3.14)	**1.89 (1.42–2.99)**
Do you chew chat				
No	174	218	1.00	1.00
Yes	36	34	1.32 (0.8–2.17)	1.94 (0.90–1.53)
Alcohol consumption				
No	74	135	1.00	1.00
Yes	136	117	2.12 (1.45–3.06)	**2.55 (1.08–2.52)**
Have friends who drink regularly				
No	149	209	1.00	1.00
Yes	61	43	1.98 (4.65–11.2)	**3.01 (2.87–3.91)**
Discussed sexual issues with parents				
No	179	118	1.00	1.00
Yes	31	134	0.15(0.73–1.80)	**0.26 (1.40–3.56)**

## Discussion

Goal five, gender equality, of sustainable development goals will not be achieved without eliminating gender based violence [5]. But sexual violence appears to be a major characteristic of school life for many young females in Ethiopia [16, 18, 19]; as it persist affecting many [20]. Most of the young people are not aware of their sexual rights and do not even appreciate the degree of their violations [21]. Thus, this study was aimed to assess sexual violence and its predictors among female Wolaita Sodo University students.

Sexual violence was found a major problem among the students with 45.4% life time prevalence. The finding was higher than the prevalence (37.3%) among female college students in Bahirdar [22]. Another study from Madawalabu University [23] revealed 41.1% life time prevalence of sexual violence which is close to this finding. However, it was lower than the prevalence among female Ambo university students 76.4% [24].

Lifetime rape was conveyed by 10.4% of respondents and it was comparable with the findings of Madawalabu and Addis Ababa Universities that revealed 10.9% [25] and 12.7% [26] rape respectively. However, it was higher than the findings from Dabat [27], Debark [28] and Addis Ababa [29] high schools students where the findings fell between 5.1% and 8.8%. This might be due to the differences in age and socio-cultural factors between study participants. The findings of attempted rape (19.2%) and rape (5.6%) in the current academic year were lower than the result from Hawassa University [30] that revealed 10.2% attempted and 3% rape.

Female students whose childhood residence was in rural areas reported higher frequency of sexual coercion. Similar findings were reported from studies among university students in Madawalabu [25] Addis Ababa [26] and Hawassa [31]. Also, study of violence that compares rural, suburban, and urban teen found teens in rural school districts to be more victims of violence than their suburban and urban counterparts [32].

Participants who had history of consuming or currently consuming alcohol and have friends who do so regularly reported higher level of sexual violence. Though, it is difficult to conclude which one comes first as the timing cannot be determined by cross sectional study; the association can be explained by various mechanisms. At a behavioral or psychological level, alcohol may decrease the risk perception and the ability to communicate assertively [33, 34], making an individual more vulnerable to sexual coercion [35]. In Ethiopia, universities and schools are alcohol free; as a result students mostly go to bars and night clubs whenever they want to drink. A previous study from Uganda suggests a causal link between alcohol consumption at bars or parties and the occurrence of sexual coercion [36]. Studies conducted in Ethiopian Universities also revealed increased risk of sexual violence among alcohol consumers [25, 30, 31]. However, it would be difficult to separate the direct effect of the alcohol consumed by the victim, from the effects associated with the setting, where the perpetrators are present [34]. Moreover, the fact that alcohol consumption and sexual assault frequently co-occur does not necessarily demonstrate that alcohol causes sexual assault; the causal direction could be the opposite. Women who report drinking could be doing so in response to having experienced sexual violence as a coping mechanism [14]. Meaning, alcohol is associated to sexual violence both as a risk and coping mechanism which makes it difficult to conclude which one comes first as the timing cannot be determined by the current study design.

Witnessing inter-parental violence or mother being beaten by her husband or male partner was associated with increased odds of sexual violence. This is also indicated in studies conducted among college students in Madawalabu [25], Hawassa [30, 31] and Chile [37] where witnessing inter-parental violence as a child increased the likelihood of experiencing sexual violence. A girl that grows observing her mother being beaten by her partner would likely believe that threats and violence are the norm in relationships [14].

Having regular boyfriend was also found to increase the risk of experiencing sexual violence. This finding is in line with study from Madawalabu University [25] where students who had a regular boyfriend experienced more sexual violence than their counterparts. As they became intimate couples might spend time in private places where the boy can force the girl for sex. As evidenced by literature, forced sex is more likely to occur later in the dating relationship than earlier [12]. Plus studies have also revealed the most frequently reported rapists to be intimate partners (husband/ boyfriend) [38, 39].

Discussing sexual issues with parents or anyone else was negatively associated with sexual violence. Students who had not reported discussion of sexual issues with anyone else showed high prevalence of sexual violence than those who did. Communication with parents on sexual topics helps young women resist partner sexual pressure [40]; as what parents have told them and might think influence their decisions about sex and relationships [41].

### Limitations of the study

Cross-sectional study cannot determine causal relationships between variables. Since sexual violence is a sensitive topic it is very prone to reporting bias, in most cases, women tend to under-report it.

## Conclusion

The high prevalence of sexual violence requires attention. More detailed research has to be conducted to develop prevention and intervention strategies.

## Additional file

Additional file 1: Annex 2: SELF-ADMINISTERED QUESTIONNAIRE (English Version). (DOCX 25 kb)
