# Supplementary material for: Sexual violence against female university students in Ethiopia
Source: BMC Int Health Hum Rights. 2017 Jul 24;17:19. doi: 10.1186/s12914-017-0127-1 (PMC5525286; doi:10.1186/s12914-017-0127-1)
Supplement: Additional file 1: — Annex 2: SELF-ADMINISTERED QUESTIONNAIRE (English Version). (DOCX 25 kb) [file 12914_2017_127_MOESM1_ESM.docx]

## Annex 2: SELF-ADMINISTERED QUESTIONNAIRE (English Version)

A Self-Administered Questionnaire Prepared to Assess the Prevalence and related factors for Sexual violence among Female Students of Wolaita Sodo University, south Ethiopia, 2015.

**INTRODUCTION:**

Now I am working on a research that tries to address social and health problems of female students in higher learning institutions*.* You have been selected to participate in the study by random selection method.

The findings of the study will be used for better understanding of the problems of female students so that it can be helpful for planning and intervention on the issue under study.

Therefore, I am requesting you to fill this questionnaire by yourself. There is no right or wrong answer in all the questions. The questions include very personal issues which may be difficult to talk about, but for many, it is found to be useful opportunity to talk.

Your response is completely confidential. You do not need to write your name and will never be used in connection with any of the information you give. You are kindly requested to answer every question and you may stop filling the form at any time you want to. However, your honest answers to these questions will help for understanding of Gender Based Violence greatly. There can be more than one answer as given on the alternative choices or opinions.

The total time needed for filling this questionnaire is about thirty minutes.

Would you be willing to participate? Yes_____ No____

If you are willing, please insert the questionnaire yourself in the sealed box around the gate of this hall after you fill it complete.

**THANK YOU!!!**

**SECTION 1: BACKGROUND CHARACTERISTICS**

| No. | **Questions (Q) and Filters** | **Answers and Alternative Choices for Responses** |
| --- | --- | --- |
| 101 | How old are you? | [____] Years |
| 102 | What is your religion? | 1. Orthodox  2. Catholic  3. Protestant  4. Muslim  8. Other (Specify) ________ |
| 103 | Where were you living before joining this college? | 1. Wolaita  2. Amhara  3. Oromiya  4. Addis Ababa  5. Tigray  8. Other (Specify)____________ |
| 104 | Where do you grow up (before age 12  where did you live longest)? | 1. Urban  2. Rural |
| 105 | What is your College/school in which you are currently learning? | ____________________ |
| 106 | What is your education level now? | 1. Year 1  2. Year 2  3. Year 3 and above |
| 107 | Have you ever been married or lived with male partner? | 1. YES  2. NO |
| 108 | Are you currently married or have boyfriend? | 1. YES, Married  2. YES, Boyfriend  3. NO _ Skip to Q 201 |
| 109 | What is the educational status of your current male partner (husband or boy friend)? | 1. No formal Education  2. Grade 1 - 8 complete  3. Grade 9 – 12 complete  4. Above grade 12  9. I don’t know |
| 110 | What is the employment status of your current male partner (husband or boy friend)? | 1. Student  2. Employed - Teacher  3. Other employee _______  4. Unemployed |

**SECTION 2: FAMILY HISTORY**

| No. | **Questions (Q) and Filters** | **Answers and Alternative**  **Choices for Responses** |
| --- | --- | --- |
| 201 | Are your father and mother living together currently? | 1. YES  2. Divorced/separated  3. Only Mother alive  4. Only Father alive  5. Both of them not alive |
| 202 | What is the educational status of your father? | 1. No formal Education  2. Grade 1 - 8 complete  3. Grade 9 – 12 complete  4. Above grade 12  9. I don’t know |
| 203 | What is the educational status of your mother? | 1. No formal Education  2. Grade 1 - 8 complete  3. Grade 9 – 12 complete  4. Above grade 12  9. I don’t know |
| 204 | When you were child, have you seen your mother being beaten by her husband or male partner? | 1. YES  2. NO |

**SECTION 3: SUBSTANCE USE**

| No. | **Questions (Q) and Filters** | **Answers and Alternative**  **Choices for Responses** |
| --- | --- | --- |
| 301 | Have you ever chewed chat? | 1. YES  2. NO _ Skip to Q 303 |
| 302 | How often do you chew chat? | 1. Every day or nearly every day  2. Once or twice a week  3. 1 – 3 times a month  4. Occasionally, less than once a month |
| 303 | Have you ever smoked tobacco/cigarette? | 1. YES  2. NO _ Skip to Q 305 |
| 304 | How often do you smoke? | 1. Every day or nearly every day  2. Once or twice a week  3. 1 – 3 times a month  4. Occasionally, less than once a month |
| 305 | Have you ever consumed alcohol (beer, areke tella, tej)? | 1. YES  2. NO _ Skip to Q 310 |
| 306 | How often do you drink alcohol (beer, areke, tella, tej)? | 1. Every day or nearly every day  2. Once or twice a week  3. 1 – 3 times a month  4. Occasionally, less than once a month |
| 307 | Have you ever been drunk in your life? | 1. YES  2. NO _ Skip to Q 310 |
| 308 | Have you been drunk since you joined this university/college? | 1. YES  2. NO |
| 309 | Have you been drunk in this academic year? | 1. YES  2. NO |
| 310 | Do you have female or male friend(s) who drink regularly? | 1. YES  2. NO_ Skip to Q 401 |
| 311 | Have you ever been used drugs or substances like cocaine? | 1. YES  2. NO |
| 312 | How often do you use drug? | 1. Every day or nearly every day  2. Once or twice a week  3. 1 – 3 times a month  4. Occasionally, less than once a month |

**SECTION 4: SEXUAL EXPERIENCES**

| No. | **Questions (Q) and Filters** | **Answers and Alternative Choices for Responses** |
| --- | --- | --- |
| 401 | Have you ever had sexual intercourse? | 1. YES  2. NO _ Skip to Q 408 |
| 402 | How old were you when you had Sexual intercourse for the first time? | 1. [_____] Years  9. I don’t know |
| 403 | How old was the person with whom you had the first sexual intercourse? | 1. [_____] Years  9. I don’t know |
| 404 | Have you been willing when you have the first sexual intercourse? | 1. YES _ Skip to Q 406  2. No |
| 405 | What was the reason for having  sexual intercourse unwillingly  (without your consent)? | 1. Family pressure/Marital engagement  2. Peer pressure  3. Threatened  4. False promise  5. For financial support (money)  6. To pass exam  7. Made me drunken  8. Other (Specify)__________ |
| 406 | Do you have more than one sexual partner currently? | 1. YES  2. NO |
| 407 | How many sexual partners have you ever experienced until now? | 1. One  2. Two  3. Three  4. Four or more |
| 408 | Can you freely discuss about your personal issues including reproductive health with your family members? | 1. YES  2. NO |

**SECTION 5: SEXUAL VIOLENCE STATUS**

Please respond genuinely to the following important questions on life events happened to you by anybody including your boyfriend or husband

| No. | **Questions (Q) and Filters** | **Before**  **Joining**  **College** | **Since**  **Joining**  **College** | **This Year** |
| --- | --- | --- | --- | --- |
|  |  | **1=YES**  **2=NO** | **1=YES**  **2=NO** | **1=YES**  **2=NO** |
| 601 | Have you ever been faced with unwelcome Touch sexually (e.g. on breasts, genitalia etc.), Verbal jocks, Comments; or made you something that you didn’t want to? | 1 2 | 1 2 | 1 2 |
| 602 | Have you ever been Forced to have sex that you have Escaped? | 1 2 | 1 2 | 1 2 |
| 603 | Have you ever been had Sexual intercourse  Forcefully or by any means that you didn’t want to or against your interest? | 1 2 | 1 2 | 1 2 |
|  |  | If nothing happened to you _  Skip to Q 701 | | |
| 604 | Who was the person who forced you for that  unwanted sex?  (More than one answer applicable) | 1. Boy friend/Husband  2. Family member  3. Other relative  4. Teacher  5. Student  6. Stranger  8. Other (specify) ______________ | | |
| 605 | How many times have you faced Forced  Sex? | 1. One time  2. Two times  3. Three times  4. Four times or more | | |
| 606 | Did you share to your family when Forced  Sex happened? | 1. YES  2. NO | | |
| 607 | Have you ever applied/reported to the legal system or police? | 1. YES  2. NO | | |
| 608 | When you faced with Forced Sex, why didn’t you share or tell to anybody?  (More than one answer applicable) | 1. I have shared/told to some body  2. Didn’t know what to do  3. Feeling of shame  4. Afraid of parents reaction  5. Afraid of the public reaction  6. Afraid of the perpetrator  8. Other (Specify) _________ | | |
| 609 | Have you ever experienced any of the  following conditions as the result of having Forced sex?  (More than one answer applicable) | 1. Unusual vaginal discharge  2. Swelling around the genitalia  3. Injury around the genitalia  4. Pregnancy  5. Abortion  8. Other (Specify) _________ | | |
| 610 | Have you ever experienced any of the following conditions as the result of having Forced Sex? (More than one answer applicable) | 1. Self blame  2. Fear  3. Anxiety  4. Hopelessness  5. Depression  6. Suicidal ideation  7. Suicidal attempt  8. Other (Specify) _________ | | |
| 611 | Have you ever experienced any of the following conditions as the result of having Forced Sex?  (More than one answer applicable) | 1. Poor Achievement/failure from school  2. Withdrawal from school  3. Rejection from family  4. Rejection from friends/peers  5. Alcohol dependency/abuse  6. Sexual dependency/abuse  7. Having multiple sexual partners  8. Other (Specify) __________________ | | |

**SECTION 6: PERCEPTIUON TOWARDS SEXUAL VIOLENCE**

| No. | **Questions (Q) and Filters** | **Answers/Choices for Responses** |
| --- | --- | --- |
| 701 | Do you think that sexual life events like  forced sex, attempting to have sex without  consent, harassing (unwelcome touches,  etc) for female college students is a  problem at this time | 1. YES  2. NO_ Skip to Q 703 |
| 702 | What do you think are the reasons for  those above mentioned problems | 1. Females’ dressing style  2. Males’ dominancy  3. Females’ low negotiation power  4. Drinking alcohol  5. Smoking cigarette  6. Chewing chat  7. Poor family control  8. Others (Specify) __________ |
| 703 | Do you think that these problems are preventable? | 1. YES  2. NO |
| 704 | What do you think are the reasons for  those above mentioned problems  (More than one answer applicable)  What do you think mechanisms for prevention of these problems?  (More than one answer applicable | 1. Legal enacting  2. Crating awareness on females  3. Creating awareness on males  4. Creating awareness on family/community  5. Empowering females  6. Strengthening clubs and associations  7. Stopping females’ appalling dressing style  8. Other (Specify) |
| 705 | Do you think those things happened on females are punishable? | 1. YES  2. NO |
| 706 | From where do you get sexuality and related information? | 1. No any source of information  2. From teachers (school)  3. From health personnel’s (health institution)  4. From family  5. From friends  6. From mass media (TV, radio, newsletters)  7. From video/film/internet/books  8. Other (Specify) _________________ |
